# Neutralization Mechanisms of Two Highly Potent Antibodies against Human Enterovirus 71

**DOI:** 10.1128/mBio.01013-18

**Published:** 2018-07-03

**Authors:** Ling Zhu, Kangwei Xu, Nan Wang, Lei Cao, Junlan Wu, Qiang Gao, Elizabeth E. Fry, David I. Stuart, Zihe Rao, Junzhi Wang, Xiangxi Wang

**Affiliations:** a National Laboratory of Macromolecules, Institute of Biophysics, Chinese Academy of Sciences, Beijing, China; b Department of Biochemistry and Molecular Biology, State Key Laboratory of Cancer Biology, the Fourth Military Medical University, Xi’an, Shanxi, China; c Division of Structural Biology, University of Oxford, Oxford, United Kingdom; d National Institutes for Food and Drug Control and WHO Collaborating Center for Standardization and Evaluation of Biologicals, Beijing, China; e Sinovac Biotech Co., Ltd., Beijing, China; Duke University School of Medicine

**Keywords:** EV71, cryo-EM, neutralizing monoclonal antibodies, viral entry, viral particle destruction

## Abstract

Despite significant advances in health care, outbreaks of infections by enteroviruses (EVs) continue to plague the Asia-Pacific region every year. Enterovirus 71 (EV71) causes hand-foot-and-mouth disease (HFMD), for which there are currently no therapeutics. Here, we report two new antibodies, A9 and D6, that potently neutralize EV71. A9 exhibited a 50% neutralizing concentration (neut_50_) value of 0.1 nM against EV71, which was 10-fold lower than that observed for D6. Investigation into the mechanisms of neutralization revealed that binding of A9 to EV71 blocks receptor binding but also destabilizes and damages the virus capsid structure. In contrast, D6 destabilizes the capsid only slightly but interferes more potently with the attachment of the virus to the host cells. Cryo-electron microscopy (cryo-EM) structures of A9 and D6 bound with EV71 shed light on the locations and nature of the epitopes recognized by the two antibodies. Although some regions of the epitopes recognized by the two antibodies overlap, there are differences that give rise to dissimilarities in potency as well as in the mechanisms of neutralization. Interestingly, the overlapping regions of the epitopes encompass the site that the virus uses to bind SCARB2, explaining the reason for the observed blocking of the virus-receptor interaction by the two antibodies. We also identified structural elements that might play roles in modulating the stability of the EV71 particles, including particle integrity. The molecular features of the A9 and D6 epitopes unveiled in this study open up new avenues for rationally designing antiviral drugs.

## IMPORTANCE

During the course of viral infections, the human body produces neutralizing antibodies which play a defining role in clearing the virus. From this study, we report two new, highly potent neutralizing antibodies, A9 and D6, against enterovirus 71 (EV71), the causative agent of HFMD. Both antibodies prevent the virus from entering the host cell, a step that is important for establishing a successful infection. A9 destabilizes and damages the virus capsid that forms an outer protective covering around the genome of the virus, while also interfering with virus attachment to the host cells. In contrast, D6 only prevents binding of the virus to its receptor(s). The mechanism of neutralization of A9 is unique and has not been observed before for neutralizing antibodies targeting EVs. The two antibodies that we are reporting in this study have potential to be developed into much-needed therapeutic interventions for treatment of HFMD, outbreaks of which are reported every year in the Asia-Pacific region.

## OBSERVATION

Enterovirus 71 (EV71), a nonenveloped single-strand RNA virus belonging to the *Picornaviridae* family, is a major causative agent of hand-foot-and-mouth disease and herpangina in children in the Asia-Pacific region (1, 2). Outbreaks of EV71 infections have also been associated with meningitis, polio-like syndrome, and encephalitis with subsequent cardiopulmonary collapse and mortality (3, 4). Currently, no antiviral therapies have been approved for treatment of EV71 infections.

EV71 virions have icosahedral symmetry and a diameter of approximately 30 nm, comprising 60 copies of the four protein subunits VP1 to VP4 (5). Each of subunits VP1 to VP3 adopts a β-barrel configuration common to many viruses (6, 7), and those subunits are arranged with icosahedral pseudo-*t* = 3 symmetry such that VP1 surrounds the 5-fold axes and VP2 and VP3 alternate about the 2- and 3-fold axes (with VP4 being internal) (8). Two types of EV71 particles, mature virus particles (termed "F-particles") and empty procapsids (termed "E-particles"), are predominantly produced during a natural infection. These two types of particles can be separated using continuous sucrose density gradient ultracentrifugation (2, 9). The E-particles have an approximately 5% larger diameter than the F-particles. Importantly, they have distinct antigenic properties that might act as baits to escape host immune system. Enteroviruses have a depression, called the “canyon,” on the viral surface encircling the 5-fold axes, which often happens to be the site of receptor binding (10). Uncoating of most enteroviruses requires expulsion of a natural lipid, also known as “the pocket factor,” from the viral capsid, generally resulting from binding of a receptor(s) into the canyon (10). Ejection of the pocket factor leads to destabilization of virions and ultimately to release of viral genomes.

**FIG 1:**
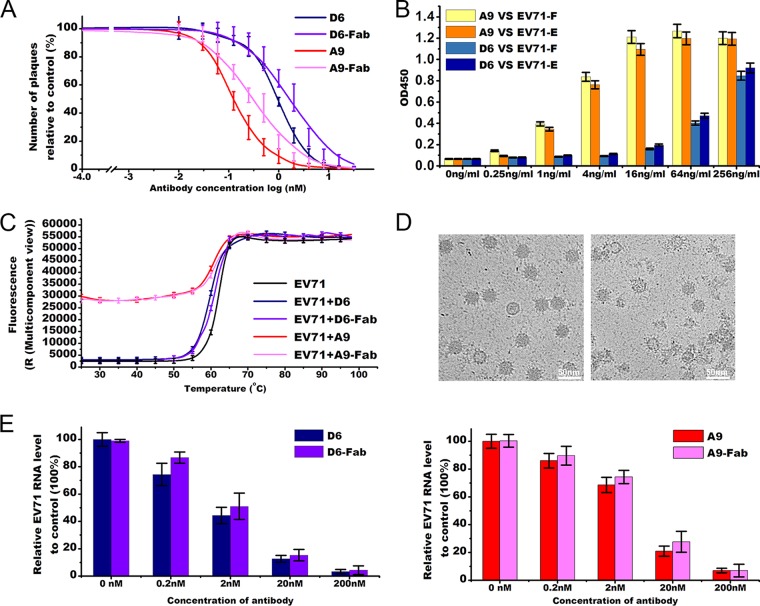
Characterizations of the D6 and A9 monoclonal antibodies. (A) Neutralization of EV71 by D6, D6-Fab, A9, and A9-Fab. D6 (blue curve), D6-Fab (purple blue curve), A9 (red curve), and A9-Fab (pink curve) were used to block EV71 infection at different concentrations by using a plaque reduction neutralization test. Data showing the levels of inhibition of virus are represented as the percentage of plaques relative to plaques in the control wells. The values represent means of results from triplicate wells with standard deviations (SD). (B) Analysis of binding of D6/A9 to EV71 full and empty particles by ELISA. Ninety-six-well plates were coated with EV71 full or empty particles, and various concentrations of D6/A9 were added. The amount of bound D6/A9 was detected by HRP assay; the parameter on the *x* axis represents the concentrations of D6/A9; the average readings of optical density at 450 nm (OD_450_) from triplicate wells at each dilution are shown with the SD. (C) The stabilities of EV71 F-particles and their complexes with D6/D6-Fab/A9/A9-Fab at neutral pH were determined by Thermofluor assay using SYTO9 dye to detect RNA exposure (12). The raw fluorescence traces are shown for EV71 F-particles (black line) as well as their complexes with D6 (blue line), D6-Fab (purple blue line), A9 (red line), and A9-Fab (pink line) following incubation with SYTO9. The values are means of results from triplicate wells with SD. (D) Cryo-EM images of EV71 particles complexed with D6-Fab (left) or A9-Fab (right) in PBS after incubation at 37°C for 10 min. (E) Amount of virus on the cell surface, as detected by RT-PCR, under conditions of exposure to D6/D6-Fab (left) or A9/A9-Fab (right) before the virus was allowed to attach to cells. High concentrations of D6/D6-Fab/A9/A9-Fab prevent attachment of EV71 to the cell surface when EV71 is exposed to D6/D6-Fab/A9/A9-Fab before cell attachment. Values represent means ± SD. Experiments were repeated in triplicate.

One of our major defense strategies against viral infections is binding of the virus by antibody (Ab), resulting in its neutralization. In the first week of acute EV71 infection, robust induction of virus-specific IgG against EV71 is detected and the response peaks in 4 to 7 days after the onset of symptoms (11). This assists in clearance of the virus. An understanding of the molecular basis of neutralization of EV71 by antibodies would aid in the development of rationally designed antiviral drugs. Working toward such a goal, we elicited two neutralizing monoclonal antibodies (NAbs), referred to as D6 and A9 here, by immunizing mice with mixtures of EV71 E- and F-particles. Both of the intact antibodies (and the Fab fragments) exhibited potent neutralizing activities against EV71, i.e., 50% neutralizing concentration (neut_50_) values of 1 nM and 0.1 nM for D6 and A9, respectively, whereas the neut_50_ values of D6-Fab and A9-Fab were found to be 3 nM and 0.4 nM, respectively (Fig. 1A). In line with the neut_50_ values, the binding affinity of A9 for EV71 was 20-fold higher than that of D6. Intriguingly, the two antibodies showed similar affinities for full and empty particles of EV71 (Fig. 1B). Neither monoclonal antibody (MAb) recognized linear epitopes in immunoblot-based assays, whilst enzyme-linked immunosorbent assay (ELISA)-based assays confirmed that they recognized conformational epitopes.

To shed light on the mechanisms of neutralization of EV71 by A9 and D6, we first investigated their effect on the stability of the virus upon binding. We performed PaSTRy assays (12) using SYTO9 dye to detect exposure of viral RNA. As with many other neutralizing antibodies, binding of D6/D6-Fab decreases the stability of EV71 F-particles only slightly. However, surprisingly, the viral RNA, which is inaccessible to fluorescent dye in EV71 F-particles and in F-particle-D6 mixtures, became accessible when F-particles were mixed with A9/A9-Fab at room temperature. This observation suggests either that the F-particles remain intact with penetrable holes, presumably in the form of uncoating intermediate capsids, or that A9/A9-Fab damages the F-particles (Fig. 1C). The same assay also indicates a two-stage transition in RNA exposure. The first event, producing a median exposure of RNA caused by A9/A9-Fab, occurs at room temperature; the second, which fully exposes the genome, occurs at higher temperature (~65°C). This is also consistent with previous studies showing that the RNA is highly packed, presumably forming a semistable structure in the EV71 virion (13). Visual inspection of the cryo-electron microscopy (cryo-EM) micrographs showed that as much as 70% of EV71 F-particles incubated with A9-Fab had been destroyed at 37°C (Fig. 1D). In contrast to this remarkable effect, only approximately 1% of the EV71 F-particles that had been incubated with D6-Fab at 37°C had altered morphologies (Fig. 1D). Interestingly, ~30% of the EV71 F-particles that had been incubated with A9-Fab at 4°C lost their RNA genome to become E-particles, while incubation of D6-Fab at 4°C seemed not alter the ratio between the EV71 F- and E-particles (see Fig. S1 in the supplemental material). In summary, A9, but not D6, destabilizes EV71 virions to induce genome release and damages EV71 particles to some extent at physiological temperatures.

10.1128/mBio.01013-18.1 FIG S1  Cryo-EM images and resolution of cryo-EM maps. (A) Cryo-EM images of EV71 particles. (B) Cryo-EM images of EV71 particles complexed with D6-Fab. (C) Cryo-EM images of EV71 particles complexed with A9-Fab. (D) The gold standard FSC curves of complexes of EV71-E-particle-D6-Fab (left) and EV71-E-particle-A9-Fab (right). (E and F) Local resolution assessment. Local-resolution EV71-E-particle-D6-Fab (E) and EV71-E-particle-A9-Fab (F) maps of density slices, rendered using ResMap (33), are shown. The red to blue color scheme corresponds to regions of relative low to high resolution. The core parts of E-particle-D6-Fab and E-particle-A9-Fab have resolution of better than 4.4 Å and 6 Å, respectively, but the resolutions of the interior parts of the maps (corresponding to the RNA genome) and most external parts are ~8 to 10 Å. Download FIG S1, JPG file, 0.8 MB. Copyright © 2018 Zhu et al. 2018 Zhu et al. This content is distributed under the terms of the Creative Commons Attribution 4.0 International license.

Note that EV71 F-particles do not start to uncoat at physiological temperatures after binding of D6. They continue to exist as mature virions and remain relatively stable like most other picornaviruses. Therefore, destabilization of the virus is unlikely to be the mechanism of neutralization by D6. To further investigate the mechanism of neutralization of EV71 by D6, real-time reverse transcription-PCR (RT-PCR) was carried out to quantify the virus remaining on the cell surface following exposure to antibodies prior to viral attachment to cells at 4°C. Both D6/D6-Fab and A9/A9-Fab could efficiently prevent attachment of EV71 to the cell surface in a concentration-dependent manner (Fig. 1E). Furthermore, D6 seemed more effective at blocking receptor attachment than A9 (Fig. 1E). The ~2 nM concentration of D6 required for blocking 50% of binding is comparable to the concentration (~1 nM) required for 50% neutralization, suggesting that this might be the main neutralization mechanism (Fig. 1E).

Given the ability of A9 to damage EV71 virions at 37°C, cryo-EM micrographs of EV71-A9-Fab and EV71-D6-Fab were prepared at 4°C and recorded using a FEI Polara electron microscope equipped with a Gatan K2 Summit detector. Image processing and three-dimensional (3D) reconstruction adopted truly independent refinements, and the resolution was assessed using the “gold standard” Fourier shell correlation (FSC) = 0.143 criterion, a good indicator of the true resolution of the map, which is as expected from theory (14). Structural characterization of F-particle-A9/D6-Fab complex might elucidate the effect of A9/D6 on the EV71 virion structure, but difficulty in obtaining homogenous complex preparations because of the structural flexibility caused by Fabs, presumably similar to the E18 MAb that triggers conformational changes on capsids (15), has made this challenging. The cryo-EM structures of E-particle-D6-Fab and E-particle-A9-Fab were determined to overall resolutions of 4.9 Å and 6.8 Å using 1,486 and 1,158 particles, respectively (Fig. S1; see also Table S1 in the supplemental material). To better analyze the interactions, the crystal structure of the EV71 E-particle was fitted into the EM maps of the complexes by CHIMERA (16). The fitting was very straightforward, with a correlation coefficient of 0.91.

10.1128/mBio.01013-18.3 TABLE S1  Cryo-EM imaging, data processing, and refinement statistics. Download TABLE S1, DOCX file, 0.02 MB. Copyright © 2018 Zhu et al. 2018 Zhu et al. This content is distributed under the terms of the Creative Commons Attribution 4.0 International license.

**FIG 2:**
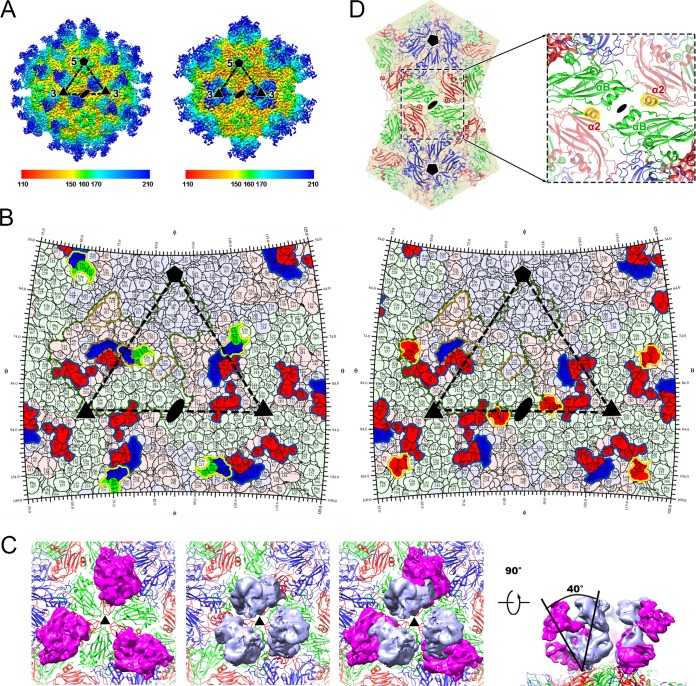
Structural features of EV71-E-particle-D6-Fab and A9-Fab. (A) The surface of cryo-EM maps of EV71-E-particle-D6-Fab (left) and A9-Fab (right), respectively. The black triangle represents an icosahedral asymmetrical unit. Five-fold and 3-fold icosahedral symmetry axes are marked "5" and "3," respectively. The reconstructions are rainbow colored in accordance with the distance (Å) of the surface from the particle center. (B) The D6 (left) and A9 (right) footprints on the EV71 surface. The figure shows a two-dimensional (2D) projection of the EV71-E-particle surface produced using RIVEM (32). Residues of VP1, VP2, and VP3 are outlined in pale blue, green, and red, respectively; residues involved in binding to D6 and A9 are shown in brighter colors corresponding to the protein chain they belong to. The footprints of D6 and A9 heavy and light chains are indicated by blue and yellow lines, respectively. The border of one protomer is indicated by a green line. Putative SCARB2 binding sites on EV71 are outlined by brown lines. Five-, 3-, and 2-fold icosahedral symmetry axes are marked as pentagons, triangles, and ovals, respectively, on one icosahedral asymmetrical unit. (C) Top views of three Fab molecules bound to EV71-E-particle. (Left) D6 Fab. (Middle) A9 Fab. (Right) Overlay of D6 (magenta) and A9 (blue) Fab molecules. (D) Cartoon representation of the atomic structure of EV71-E-particle, showing two neighboring icosahedral pentamers. Blue, VP1; green, VP2; red, VP3. The inset shows the 2-fold-symmetry-related helices, and the VP3 α2 helices involved in binding to A9 are highlighted in yellow.

A total of 60 copies of D6-/A9-Fabs bound to each particle (Fig. 2A), around the edges of the pentameric building blocks of the virus—specifically, between the 2-fold and 3-fold axes (Fig. 2A). This binding region is broadly similar in location to those observed previously for R10 antibody bound to hepatitis A virus and NAb E18 bound to EV71 (15, 17). However, while three A9-Fab molecules formed a compact cluster around the 3-fold axes, the Fabs of D6 were comparatively more outspread (Fig. 2A). The entire binding site of the Fab fragment of D6 was located within a protomer, primarily comprising the VP3 “puff,” VP3 BC loop, VP2 GH loop, and VP1 C terminus (residues 280 to 292) (Fig. 2B). Among these, the VP3 BC loop and VP1 C terminus are in close proximity to the “canyon” and have been implicated in SCARB2 binding (10). The overlapping footprint of the A9-Fab spanned the region between the 2-fold and the 3-fold axes, including VP3 “puff,” the VP3 BC loop, VP3 α2 (residues 144 to 150), and the VP1 C terminus (residues 289 to 294), despite the top view of three A9-Fabs showing a “trimer”-like structure around the 3-fold axes (Fig. 2B and C). Superimposition of the cryo-EM structures of the two antibodies bound to EV71 defined the regions of overlap of the binding sites of the two MAbs, which include the putative binding site for SCARB2. This structural observation is consistent with the results of the functional assays, which revealed that both D6 and A9 blocked the attachment of EV71 to the host cells. The D6-Fabs and A9-Fabs adopted distinct configurations upon binding to the virion (Fig. 2C), with the A9-Fabs being rotated toward each other by ~40° compared to D6, resulting in the far ends of the three A9-Fabs coming close to each other, giving rise to a “trimer”-like structure of Fabs anchored on the surface of EV71 (Fig. 2C). A9 IgG avidity is likely to be a consequence of the two Fab arms of an IgG on the surface of EV71 being sufficiently close to support bivalent attachment.

Picornavirus entry can be subdivided into two key steps: (i) attachment, in which the virus binds to its cellular receptors on the surface of the host cell, and (ii) uncoating, in which the viral genome is released from viral particles into host cells (18). Neutralization by D6 is mediated by inhibition of EV71 attachment to host cells, possibly via blocking the binding of the virus to the receptor by steric hindrance. In contrast to D6 and other previously reported neutralizing antibodies against EV71, A9 destabilizes and, to some extent, disrupts the virus quaternary structure at physiological temperatures before the virus can enter host cells. Less-violent mechanisms of inhibition, such as destabilization of the virion structure and initiation of viral genome release by NAbs such as E18 (anti-EV71) and R10 (anti-hepatitis A virus [HAV]), which mimic cell receptors, are relatively common neutralizing mechanisms in picornaviruses (15, 17, 19). In contrast, NAbs destabilizing and disrupting viral particles tend to target the regions located adjacent to the icosahedral 2-fold axes, which separate during the initial stage of uncoating in enteroviruses (2, 9). In comparison to NAbs inhibiting EV71 infection via particle destabilization, an additional epitope of A9 on EV71 maps to the VP3 α2 helix. A superposition of the EV71-E-particle-A9-Fab structure onto the EV71-F-particle structure shows notable structural shifts of the VP3 α2 helices and the VP2 αA helices at icosahedral 2-fold axes, which possibly contribute to the ability of A9 to disrupt EV71 F-particles (Fig. S2). Since the α2 helices of VP3 together with αA helices of VP2 surround the icosahedral 2-fold axes, these interactions play a role in modulating stability of the particles, including particle integrity (Fig. 2D). The role of these regions in controlling stability is confirmed by the observation that they can be targeted to overstabilize the viral capsid, for example, by capsid engineering for improved vaccines (20, 21). The molecular features of the D6 and A9 epitopes identified in this study reveal the receptor recognition sites and structural elements modulating viral stability and particle integrity, opening new avenues and targets for structure-based rational antiviral drug design. The results of our studies also highlight the promise of using antibody-based therapeutic interventions for the treatment of acute EV71 infections, for which there are currently no FDA-approved drugs.

10.1128/mBio.01013-18.2 FIG S2  Structural comparison of two neighboring icosahedral pentamers from the EV71-E-particle-A9-Fab and the uncomplexed EV71-F-particle. Capsid proteins VP1, VP2, and VP3 from E-particle-A9-Fab are colored in blue, green, and red, respectively. All capsid proteins from the uncomplexed F-particle are colored in wheat. The A9 epitope on EV71-E-particle is shown as spheres. Insets show the major structural differences. Download FIG S2, JPG file, 0.9 MB. Copyright © 2018 Zhu et al. 2018 Zhu et al. This content is distributed under the terms of the Creative Commons Attribution 4.0 International license.

### Particle production and purification.

EV71 genotype C4 was produced in Vero cells at a multiplicity of infection (MOI) of 0.2 at 37°C. Particle production and purification have been described previously (2). All animal procedures were carried out in accordance with the guideline for the Use of Animals in Research issued by the Institute of Biophysics, Chinese Academy of Sciences.

### Production of Fab fragments.

D6 and A9 were purified from mouse ascites with a protein A affinity column (GE). The Fab fragment was generated using a Pierce FAB preparation kit (Thermal Scientific), according to the manufacturer’s instructions. Briefly, after removal of the salt using a desalting column, antibody was mixed with papain and then digested at 37°C for 6 h. The Fab fragment was separated from the Fc fragment by using a protein A affinity column. Then, Fab was loaded onto a Superdex 200 gel filtration column (GE). Fractions corresponding to the major peak were collected and concentrated for cryo-EM analysis.

### Cryo-EM and data collection.

Purified D6 and A9 Fab fragments were incubated with purified EV71 (at a concentration of ~1 mg/ml) separately on ice for 10 min at a ratio of 120 Fab molecules per virion. A 3-µl aliquot of the mixtures of EV71-D6 and EV71-A9 was transferred onto a freshly glow-discharged 400-mesh holey carbon-coated copper grid (C-flat, CF-2/1-2C; Protochips). Grids were blotted for 3 s in 90% relative humidity for plunge-freezing (Vitrobot; FEI) in liquid ethane. Cryo-EM data sets were collected at 300 kV using an FEI Tecnai G2 Polara microscope (FEI) equipped with a K2 detector. Movies (25 frames, each 0.2 s, total dose 25 e^−^ Å^−2^) were recorded with a defocus of between 1 and 2.5 µm using SerialEM (22), which yields a final pixel size of 1.35 Å.

### Image processing, 3D reconstruction, and model refinement.

The micrographs from each movie were aligned and the corresponding data averaged for the correction of beam-induced drift using MOTIONCORR (23). Good particles from micrographs were picked manually using the boxer program in the EMAN (24) package. The contrast transfer function (CTF) parameters for each micrograph were estimated by using a graphics processing unit (GPU) accelerated program, Gctf (25). The structures were determined using Relion (26), with icosahedral symmetry applied. Totals of 1,486 and 1,158 particles for EV71-E-particle-D6-Fab and EV71-E-particle-A9-Fab were used to obtain the final density maps at 4.9 Å and 6.8 Å, respectively (the initial models were created by EMAN2 [27]), as evaluated by Fourier shell correction (threshold = 0.143 criterion) (14). The crystal structures of EV71-E-particle (2) and the R10 Fab (PDB identifier [ID] 5WTF [17]) were fitted into cryo-EM maps of EV71-E-particle-D6-Fab and EV71-E-particle-A9-Fab complexes using the program CHIMERA (16). The fitted models were reﬁned against the structure factors obtained by Fourier back-transforming the cryo-EM map using Phenix (28). Further improvement was achieved by iterative cycles of manual model rebuilding and pseudocrystallographic refinement (29). Briefly, obtained phase probabilities, written in the form of Hendrickson-Lattman (HL) coefficients, were used for reciprocal space refinement, performed against an MLHL (maximum likelihood with experimental phase probability distribution) target in Phenix (28) using both X-ray and electron scattering factors (29). To fulfill the requirements of the crystallographic MLHL refinement, 5% of the reflections were selected randomly for the “Rfree” set, the components of which were kept identical for all refinements. The quality of the model was verified by visual inspection and by calculating the crystallographic working and free R factors in Phenix (28).

### Plaque reduction neutralization test.

Different concentrations of D6/D6-Fab or A9/A9-Fab were incubated at a 1:1 ratio by volume with infectious EV71 (300 PFU/ml) for 1 h at 37°C. These virus-antibody mixtures were used to inoculate monolayers of Vero cells cultured in 24-well plates in triplicate. The monolayers contained 3 × 10^5^ Vero cells/ml grown in 0.5 ml of Dulbecco’s modified Eagle’s medium (DMEM) supplemented with 5% fetal bovine serum (FBS). An overnight incubation resulted in adherent cultures, which were used for the subsequent studies. The medium was aspirated from the adherent cultures before inoculation with 200 µl of the antibody-virus mixtures was performed. The plates were incubated in a CO_2_ incubator at 37°C for 2 h, after which 1 ml of DMEM supplemented with 2% FBS and 1.5% carboxymethyl cellulose was overlaid in each well. Plates were incubated at 37°C with 5% CO_2_ for 4 days and stained with naphthalene black. Plaques were counted manually. Percentages of inhibition of infection were estimated by comparing the number of plaques observed for wells inoculated with antibody-virus mixtures with those of controls inoculated with the virus only.

### RT-PCR to quantitate virus on cell surface.

The amount of EV71 remaining on the surface of Vero cells after D6/D6-Fab or A9/A9-Fab treatment was estimated by quantitative RT-PCR, as described previously (17). Briefly, EV71 was mixed with different concentrations of D6/D6-Fab or A9/A9-Fab before attachment of the virus to Vero cells (MOI = 1) at 4°C. The cells were washed with phosphate-buffered saline (PBS) three times, and total cellular RNA was purified using an RNeasy minikit (Qiagen) according to the manufacturer’s instructions. Real-time quantitative PCR was performed using a One Step SYBR PrimeScript RT-PCR kit (TaKaRa) in a CFX 96 real-time system (Bio-Rad). The 25 µl of reaction buffer contained 12.5 µl 2× One Step SYBR RT-PCR buffer III, 0.5 µl *Ex Taq* HS, 0.5 µl PrimeScript RT enzyme mix II, 0.5 µl forward primers (5′ GTCCTTAATTCGCACAGCACAGCT 3′), 0.5 µl reverse primers (5′ CGGTCCGCACTGAGAATGTACCCAT 3′), 2 µl total RNA, and 8.5 µl RNase-free H_2_O. The thermal profile for RT-PCR was 42°C for 5 min for reverse transcription and then 95°C for 10 s for inactivating reverse transcription polymerase, followed by 40 cycles of denaturation at 95°C for 10 s and annealing and, finally, extension at 60°C for 30 s. β-Actin was used as the housekeeping gene to normalize variance between samples (forward, 5′ GGCCAGGTCATCACCATT 3′; reverse, 5′ ATGTCCACGTCACACTTCATG 3′). The analysis of relative levels of EV71 RNA in different samples was performed by the comparative threshold cycle (2^−ΔΔ*CT*^) method (30).

### Thermofluor assay.

Thermofluor experiments were performed with an MX3005p RT-PCR instrument (Agilent). SYTO9 (Invitrogen) was used in the form of fluorescent probes to detect the presence of single-stranded RNA (31). Fifty-microliter reactions were set up in a thin-walled PCR plate (Agilent), using reaction mixtures containing either 1.0 µg of virus or 1.0 µg of virus plus 3.0 µg of D6/D6-Fab/A9/A9-Fab (~120 antibody molecules per HAV virion) and 5 µM SYTO9 in PBS solutions. The temperature was ramped from 25°C to 99°C, with fluorescence recorded in triplicate at 1°C intervals. The RNA release (Tr) data were taken as the minima of the negative first derivative of the RNA exposure.

### Virus-MAb binding affinity measurement.

The binding affinity of EV71 (full or empty particles) to purified D6 or A9 was measured by direct ELISA. Briefly, 96-well plates were coated with 100 µl per well of inactivated EV71 full or empty particles (5 µg/ml in PBS buffer) overnight at 4°C. The wells were then incubated sequentially with 100 µl per well of 5% (wt/vol) bovine serum albumin (BSA)–PBST (PBS with 0.2% Triton X-100) at 37°C for 1 h and 100 µl per well of D6/A9 at various concentrations at 37°C for 1 h. Horseradish peroxidase (HRP)-conjugated goat anti-mouse IgG (Abnova) diluted (1:5,000) in PBST plus 1% BSA was used as the secondary antibody at 37°C for 1 h. Extensive washes with PBST were carried out between incubation steps. For color development, 100 µl per well of 3,3′,5,5′-tetramethylbenzidine (TMB) was added, the reaction mixture was incubated at 37°C for 10 min, and the reaction was stopped by the addition of 50 µl of 1 M H_3_PO_4_ per well. Absorbance was measured at 450 nm in a 96-well plate reader.

### Accession number(s).

Cryo-EM density maps of EV71-E-particle-D6-Fab and EV71-E-particle-A9-Fab have been deposited in the Electron Microscopy Data Bank under accession numbers EMD-6963 and EMD-6964, respectively. The fitted atomic coordinates for EV71-E-particle-D6-Fab and EV71-E-particle-A9-Fab have been deposited in the Protein Data Bank with code accession numbers 5ZUD and 5ZUF, respectively.
