# Supplementary figures and images for: Neutralization Mechanisms of Two Highly Potent Antibodies against Human Enterovirus 71
Source: mBio. 2018 Jul 3;9(4):e01013-18. doi: 10.1128/mBio.01013-18 (PMC6030555; doi:10.1128/mBio.01013-18)

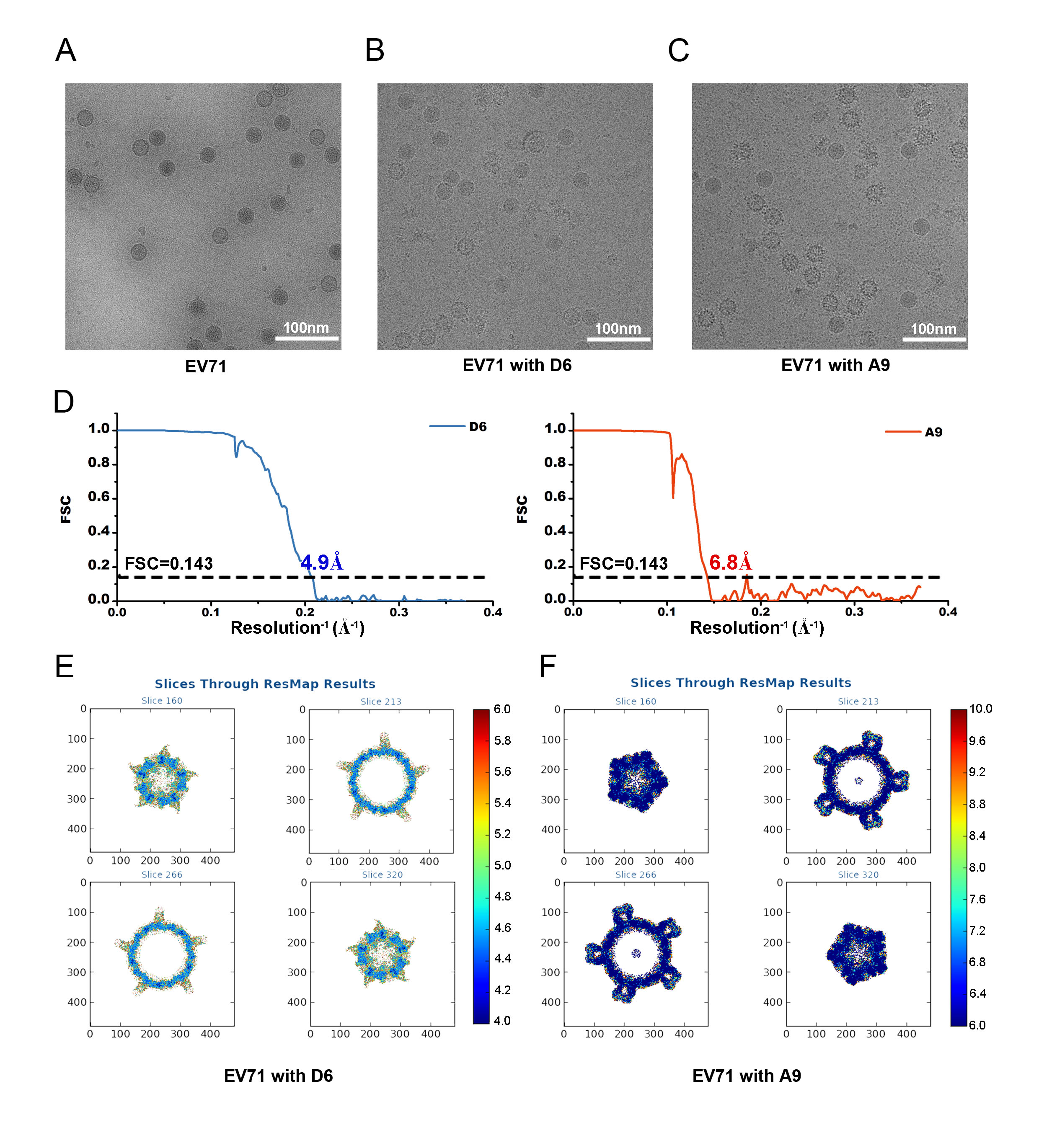

Supplement: FIG S1 [file mbo004183967sf1.jpg]

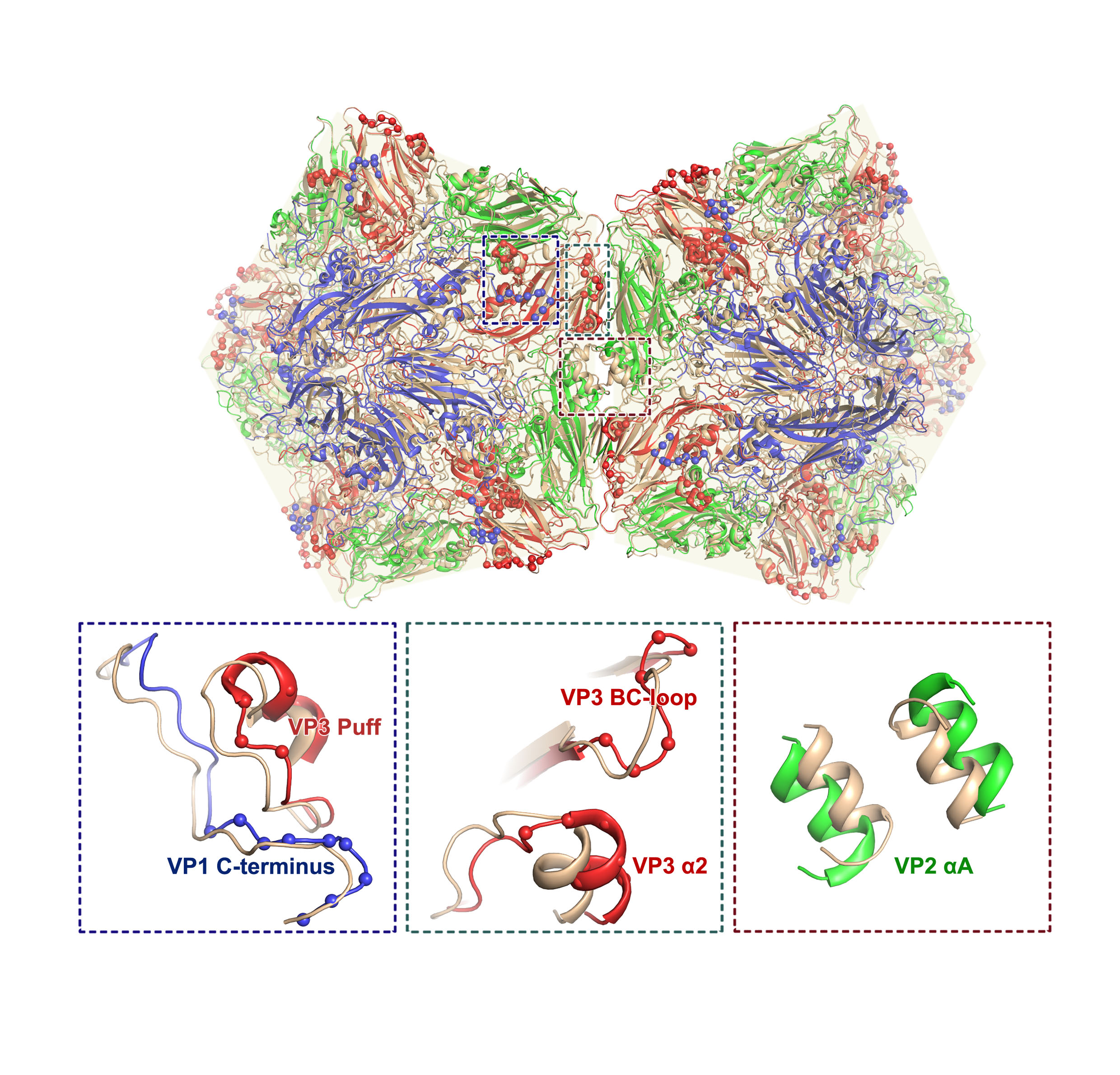

Supplement: FIG S2 [file mbo004183967sf2.jpg]
