# Supplementary material for: Neutralization Mechanisms of Two Highly Potent Antibodies against Human Enterovirus 71
Source: mBio. 2018 Jul 3;9(4):e01013-18. doi: 10.1128/mBio.01013-18 (PMC6030555; doi:10.1128/mBio.01013-18)
Supplement: TABLE S1 [file mbo004183967st1.docx]

**Table. S1**

**Cryo-EM imaging, data processing and refinement statistics.**

| Name | EV71-E-particle-D6-Fab | | EV71-E-particle-A9-Fab | |
| --- | --- | --- | --- | --- |
| **Data collection** | |  | |  |
| Micrographs (total) | | 194 | | 188 |
| Micrographs (used) | | 158 | | 123 |
| Particles selected | | 1,766 | | 1,388 |
| Particles included in final reconstruction | | 1,486 | | 1,158 |
| Sampling, Å per pixel | | 1.35 | | 1.35 |
| Defocus range (μm) | | 1.2-2.5 | | 1.4-2.8 |
| Resolution (Å) (FSC = 0.143 criterion) | | 4.9 | | 6.8 |
| **Models Refinement** | |  | |  |
| Clashscore | | 26.7 | | 29.8 |
| Rotamer outliers (%) | | 0.38 | | 0.54 |
| C-beta deviations | | 0 | | 0 |
| Ramachandran statistics (%) | |  | |  |
| Most favored | | 90.8 | | 90.0 |
| Allowed | | 7.3 | | 7.8 |
| Outliers | | 1.9 | | 2.2 |
| R.m.s.deviations | |  | |  |
| Bond lengths (Å) | | 0.012 | | 0.011 |
| Bond angles (°) | | 1.166 | | 1.068 |
| *R*_work_/*R*_free_ (%) | | 35.7/36.2 | | 36.8/37.1 |
